# Plasma half-life and tissue distribution of leukocyte cell-derived chemotaxin 2 in mice

**DOI:** 10.1038/s41598-020-70192-x

**Published:** 2020-08-06

**Authors:** Akihiro Kikuchi, Hiroaki Takayama, Hirohiko Tsugane, Kazuhiro Shiba, Keita Chikamoto, Tatsuya Yamamoto, Seiichi Matsugo, Kiyo-aki Ishii, Hirofumi Misu, Toshinari Takamura

**Affiliations:** 1 Department of Endocrinology and Metabolism, Kanazawa University Graduate School of Medical Sciences, Kanazawa, 920-8640 Japan; 2 Division of Endocrinology and Metabolism, Department of Homeostatic Regulation, National Institute for Physiological Sciences, National Institute of Natural Sciences, Okazaki, 444-8585 Japan; 3 Life Sciences Division, Engineering and Technology Department, Kanazawa University, Kanazawa, 920-8640 Japan; 4 Division of Natural System, Graduate School of Natural Science and Technology, Kanazawa University, Kanazawa, 920-1192 Japan; 5 Advanced Science Research Center, Kanazawa University, Kanazawa, 920-8640 Japan; 6 Suntory Foundation for Life Sciences, Bioorganic Research Institute, Kyoto, 619-0284 Japan; 7 Department of Integrative Medicine for Longevity, Graduate School of Medical Sciences, Kanazawa University, Kanazawa, 920-8640 Japan

**Keywords:** Obesity, Nutrition

## Abstract

Leukocyte cell-derived chemotaxin 2 (LECT2) is a hepatokine that causes skeletal muscle insulin resistance. The circulating levels of LECT2 are a possible biomarker that can predict weight cycling because they reflect liver fat and precede the onset of weight loss or gain. Herein, to clarify the dynamics of this rapid change in serum LECT2 levels, we investigated the in vivo kinetics of LECT2, including its plasma half-life and tissue distribution, by injecting ^125^I-labelled LECT2 into ICR mice and radioactivity tracing. The injected LECT2 was eliminated from the bloodstream within 10 min (approximate half-life, 5 min). In the kidneys, the radioactivity accumulated within 10 min after injection and declined thereafter. Conversely, the radioactivity in urine increased after 30 min of injection, indicating that LECT2 is mainly excreted by the kidneys into the urine. Finally, LECT2 accumulated in the skeletal muscle and liver until 30 min and 2 min after injection, respectively. LECT2 accumulation was not observed in the adipose tissue. These findings are in agreement with LECT2 action on the skeletal muscle. The present study indicates that LECT2 is a rapid-turnover protein, which renders the circulating level of LECT2 a useful rapid-response biomarker to predict body weight alterations.

## Introduction

Obesity is considered a risk factor for diseases, including type 2 diabetes and non-alcoholic fatty liver disease (NAFLD)^1, 2^. Current treatments comprising dieting and physical exercises cannot always achieve adequate weight reduction in the long term because of body weight cycling, *i.e.* intentional loss and unintentional regaining of weight^3^. Body weight cycling may be associated with subsequent weight regain^4^ and development of insulin resistance^5^. To achieve appropriate weight control in obese individuals, rapid-response biomarkers that can predict body weight alterations are desirable to facilitate early clinical interventions. Additionally, such biomarkers encourage individuals to lose weight. In humans, serum levels of the continually synthesised and degraded secretory proteins can be utilised as sensitive nutritional parameters^6^. Rapid-turnover proteins, such as transferrin, prealbumin and retinol-binding protein (RBP), are used for the assessment of additional nutritional support in hospitalised patients^7, 8^. The serum half-life of RBP is 0.5 days; therefore, it reflects real-time nutrition status^9^. However, an increase in the RBP levels is not always a representative of overnutrition or weight regain. There is no significant correlation between RBP levels and body mass index (BMI); however, an increase in the RBP levels has been reported in people with obesity and type 2 diabetes^10^. To the best of our knowledge, no established indicators for conditions of overnutrition and obesity have been reported to date.

Leukocyte cell-derived chemotaxin 2 (LECT2) is a liver-derived secretory protein that was initially identified as a chemotactic factor for neutrophils^11^. LECT2 is a modulator of immune and inflammatory reactions. Lu et al. showed that LECT2 treatment in septic mice improves protective immunity by enhancing macrophage function^12^. Furthermore, Saito et al. showed that LECT2 might play an essential role in the pathogenesis of hepatitis by modulating natural killer T-cells in the liver^13^, whereas Anson et al. showed that LECT2 plays a critical role in anti-inflammatory and tumour-suppressive pathways in β-catenin-induced liver tumorigenesis^14^. Xu et al. recently showed that LECT2 is a functional ligand for the orphan receptor Tie1 and that LECT2/Tie1 signalling promotes liver fibrogenesis^15^. We rediscovered LECT2 as a hepatokine involved in metabolic disorders. LECT2 impairs insulin action by activating c-Jun N-terminal kinase (JNK) in the skeletal muscle^16^. In addition, it reduces energy expenditure by impairing myogenesis^16^. Therefore, LECT2 induces insulin resistance in the skeletal muscle and sarcopenic obesity observed in obese individuals with type 2 diabetes. Identification of the LECT2 receptor in the skeletal muscle can provide detailed insights into the mechanisms linking obesity with the pathogenesis of insulin resistance in the skeletal muscle, which facilitates the development of LECT2-targeted agents for the treatment of insulin resistance.

Hepatic *LECT2* expression positively correlates with BMI and waist circumference in humans^16^. The plasma levels of LECT2 are higher in individuals with type 2 diabetes^17^ and in those with NAFLD compared with those in controls^18^. Serum LECT2 levels predict diet-induced body weight cycling in mice^19^. LECT2 levels are rapidly elevated prior to weight gain, while these are rapidly reduced prior to weight loss^19^. The hepatic expression of LECT2 is negatively regulated by an energy depletion-sensing protein adenosine monophosphate-activated protein kinase (AMPK)^16^. Indeed, serum LECT2 levels reflect liver fat content rather than adipose tissue mass. These features of LECT2 may explain why its serum levels sensitively reflect weight cycling. In addition, we reported that aerobic exercise for 3 h reduces serum LECT2 levels in mice^16^, suggesting that not only diet therapy but also exercise therapy reflect serum LECT2 levels. Considering the rapid response of circulating LECT2 linked to subsequent body weight alterations, the measurement of serum LECT2 levels might be a clinically useful indicator of overnutrition.

The quantification of LECT2 to elucidate its protein kinetics is a key factor for its development as a clinical biomarker of overnutrition conditions. Radioactive labelling with ^125^I is one of the techniques commonly utilised to determine the in vivo kinetics of proteins^20^. Although various procedures have been used to introduce ^125^I into proteins, the chloramine-T method is a well-established method that covalently links ^125^I to the tyrosine residues of the target protein^21^. Based on the crystal structure of human LECT2, tyrosine residues are present on the surface of the protein^22^, suggesting the possibility of its successful iodination using the chloramine-T method.

In the current study, we quantified the in vivo protein kinetics of LECT2 in ICR mice using a recombinant murine ^125^I-labelled LECT2 protein.

## Results

### LECT2 expression, purification and iodination

**Figure 1:**
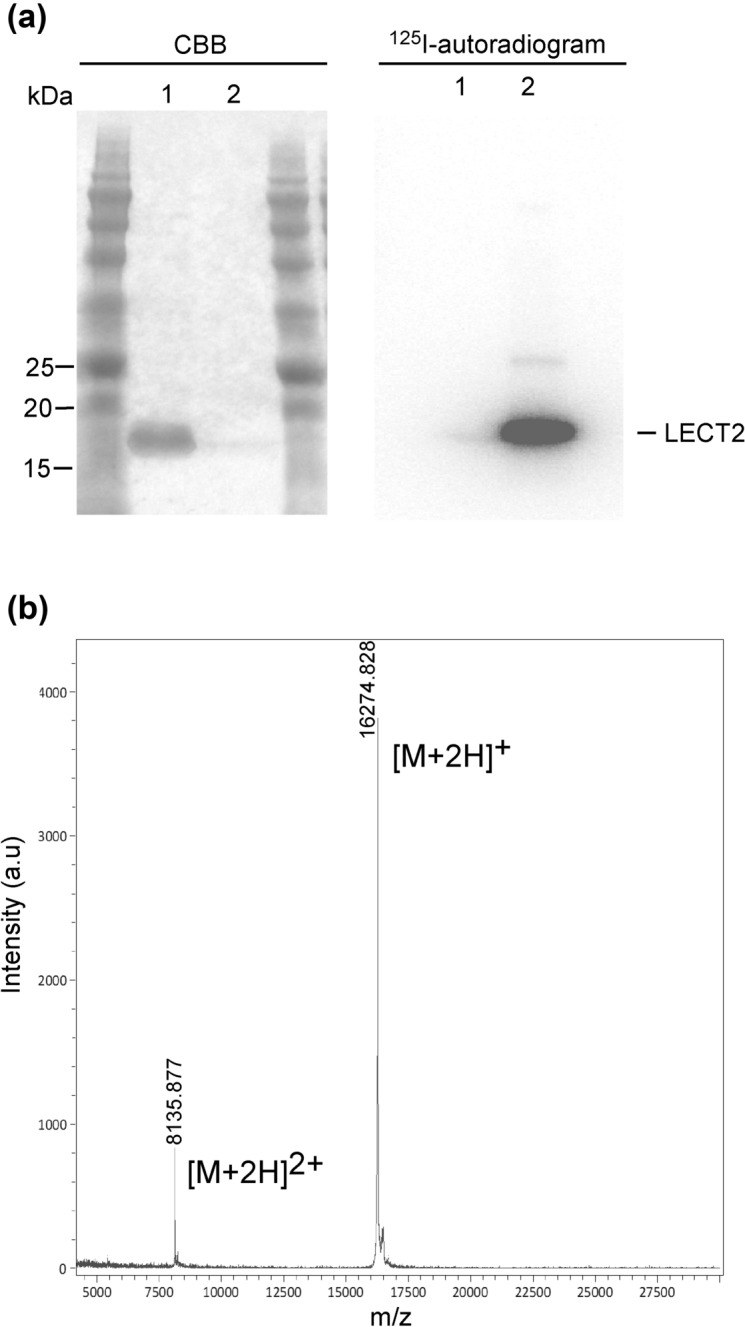
(**a**) Sodium dodecyl sulphate–polyacrylamide gel electrophoresis analysis of the purified murine leukocyte cell-derived chemotaxin 2 (LECT2). CBB staining (left) and ^125^I-autoradiogram (right). Lane 1: non-labelled LECT2. Lane 2: ^125^I-labelled LECT2. (**b**) Mass spectrum of the purified murine LECT2 by matrix‐assisted laser desorption/ionisation. The full-length gels are presented in Supplementary Fig. 1.

We cloned cDNA encoding murine *Lect2* into the pCMV-Myc-C vector. The recombinant protein with the isoelectric point (pI) of 9.0 was expressed in Expi293 cells and purified by cation exchange chromatography, followed by c-Myc affinity chromatography. The purified protein was observed as a single band on sodium dodecyl sulphate–polyacrylamide gel electrophoresis (Fig. 1a, left panel). In addition, the mass spectrum of the protein by matrix‐assisted laser desorption/ionisation was 16,274.8 Da (Fig. 1b). Since the mass calculated from the amino acid sequence of the secreted form of murine LECT2 (without signal sequence) is 16,280.48 Da, the recombinant protein had no post-translational modifications such as glycosylation and formed three disulphide bonds as reported^23^.

Radioactive ^125^I was incorporated into the purified LECT2 using the chloramine-T method. The iodination did not alter electrophoretic mobility (Fig. 1a, right panel), indicating that the protein did not undergo denaturation during iodination.

### Circulating LECT2 levels in ICR mice

**Figure 2:**
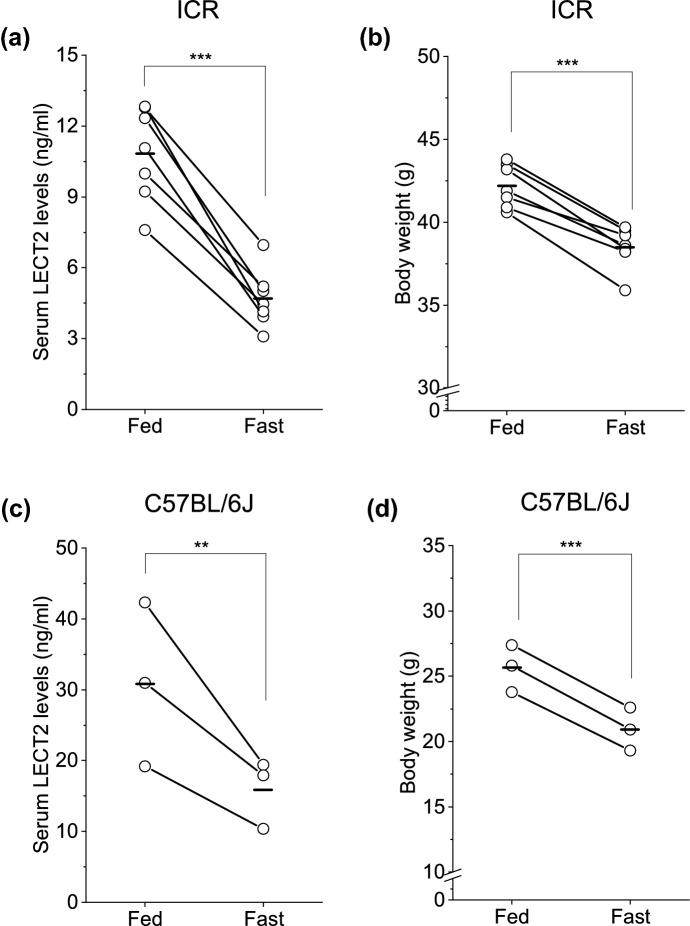
(**a**) Serum levels of LECT2 and (**b**) body weight in ICR mice before and after 17 h-fasting (n = 7). (**c**) Serum levels of LECT2 and (**d**) body weight in C57BL/6J mice before and after 24 h of fasting (n = 3). **P < 0.01, ***P < 0.001.

To confirm the rapid response of LECT2 levels in ICR mice, we fasted male mice for 17 h. Endogenous LECT2 levels decreased after fasting with weight reduction (Fig. 2a,b); this result was consistent with that of our previous report using C57BL/6 J mice^16^ (Fig. 2c,d).

Next, we evaluated whether not only endogenous LECT2 but also the recombinant protein injected via the tail vein were eliminated from the bloodstream. The C57BL/6 J mice were fasted for 16 h followed by injection of 0 (phosphate-buffered saline [PBS]), 100 and 1,000 ng non-labelled LECT2 per mouse. After the injection, mice were fasted for another 24 h. The serum LECT2 levels were 10.5 ± 5.0, 11.5 ± 3.4 and 11.4 ± 4.8 ng/mL in mice injected with 0, 100 and 1,000 ng LECT2, respectively. Thus, not only endogenous LECT2 but also the recombinant LECT2 protein was eliminated from the bloodstream of mice.

### In vivo kinetics of LECT2 in mice

**Figure 3:**
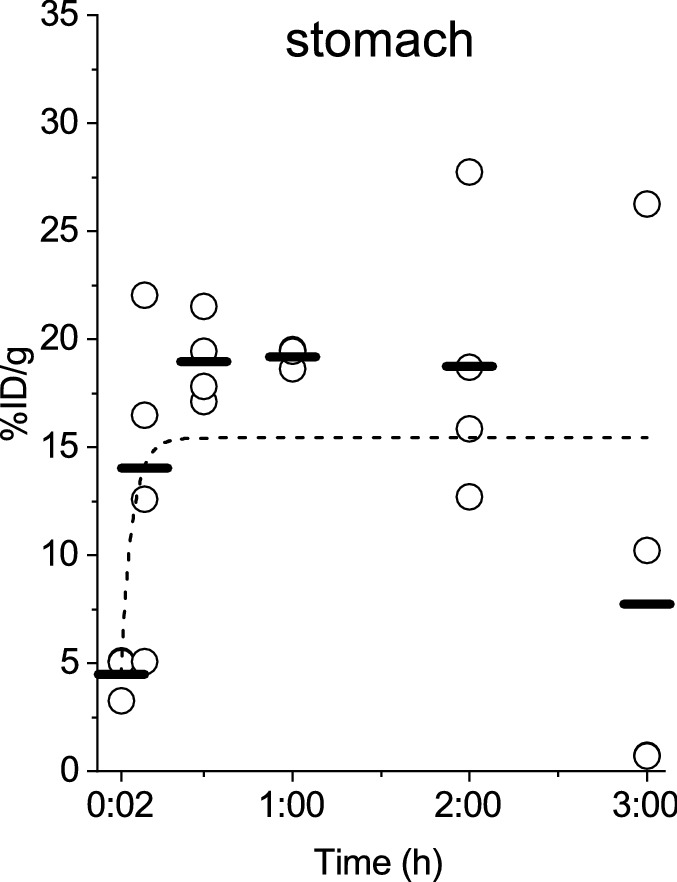
Percentage of the injected dose per gram of tissue (%ID/g) in the stomach at 2 min, 10 min, 30 min, 1 h, 2 h and 3 h after ^125^I-LECT2 injection. There was no significant difference among the time points.

**Figure 4:**
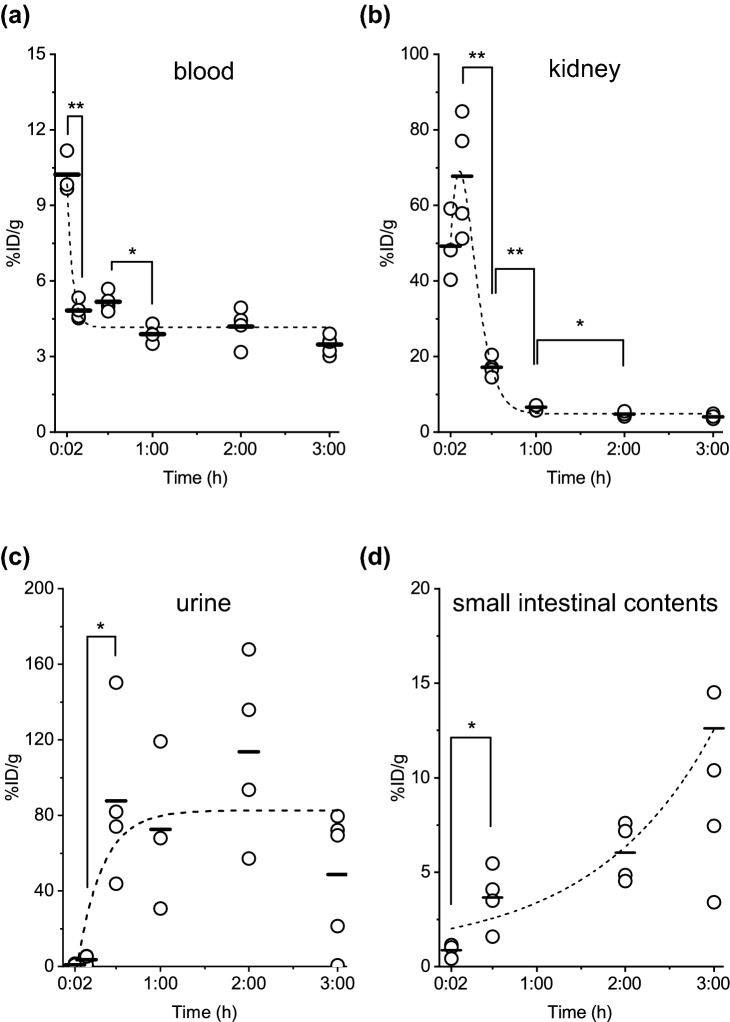
Percentage of the injected dose per gram of tissue (%ID/g) in the (**a**) blood, (**b**) kidney, (**c**) urine and (**d**) small intestinal contents at 2 min, 10 min, 30 min, 1 h, 2 h and 3 h after ^125^I-LECT2 injection. *P < 0.05, **P < 0.01.

**Figure 5:**
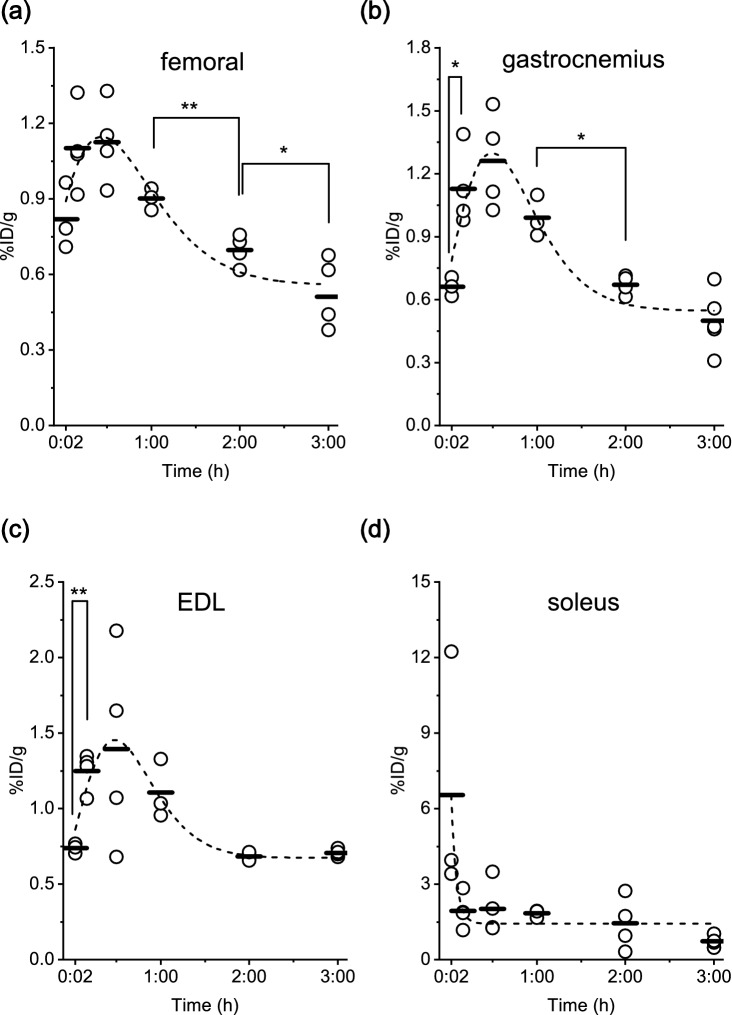
Percentage of the injected dose per gram of tissue (%ID/g) in the (**a**) femoral muscle, (**b**) gastrocnemius muscle, (**c**) extensor digitorum longus muscle and (**d**) soleus muscle at 2 min, 10 min, 30 min, 1 h, 2 h and 3 h after ^125^I-LECT2 injection. *P < 0.05, **P < 0.01.

**Figure 6:**
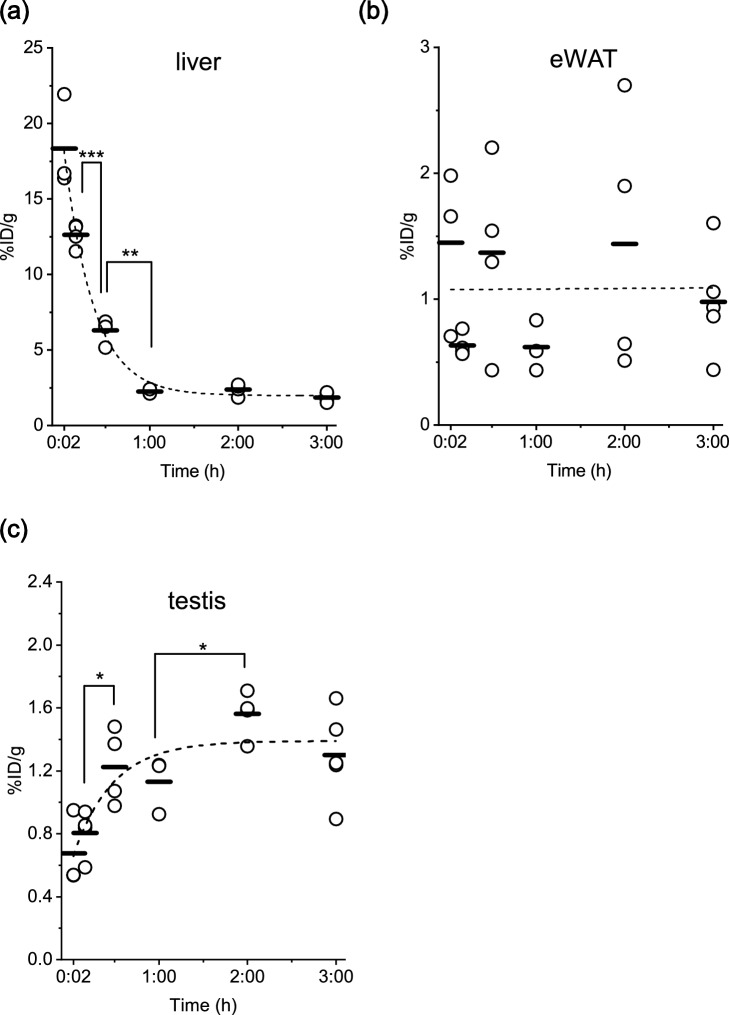
Percentage of the injected dose per gram of tissue (%ID/g) in the (**a**) liver, (**b**) epididymal adipose tissue (eWAT) and (**c**) testis at 2 min, 10 min, 30 min, 1 h, 2 h and 3 h after ^125^I-LECT2 injection. *P < 0.05, **P < 0.01, ***P < 0.001.

To investigate the in vivo kinetics of LECT2 in mice, we injected ^125^I-labelled LECT2 to overnight-fasted ICR mice via the tail vein. The mice were sacrificed at 2 min (n = 3), 10 min (n = 4), 30 min (n = 4), 1 h (n = 3), 2 h (n = 4), and 3 h (n = 5) post-injection of the tracer (1.21 μg/body LECT2 which was prepared by mixing approximately 33 kBq ^125^I-LECT2 (0.01 μg) and non-labelled LECT2 (1.2 μg)). Various organs and tissues as well as urine, blood, and small intestinal contents were collected for the measurement of radioactivity by the gamma counter to calculate the percentage of the injected dose per gram of tissue (%ID/g) (Fig. 3, 4, 5, 6). Moreover, we investigated the in vivo kinetics of bovine serum albumin (BSA) to compare the kinetics of the two proteins (Supplementary Fig. 2–5 and Supplementary Table 1).

### Evaluation of in vivo deiodination of ^125^I-LECT2

The in vivo deiodination of ^125^I-LECT2 may contribute to the localisation of radioactivity in the tissues^24^. The biodistribution study of free ^125^I in rats revealed that the radioactivity was increased in the stomach up to 6 h after injection ^25^. Conversely, the injection of ^125^I-LECT2 to ICR mice showed that the radioactivity increased for only the first 30 min in the stomach, implying partial in vivo deiodination of ^125^I-LECT2. However, there was no change in the percentage of the injected dose per gram of tissue in the stomach after 30 min (Fig. 3), which was quite different from the reported biodistribution of ^125^I in rats^25^. Thus, the tissue radioactivity levels in the current study primarily reflected the accumulation of LECT2 protein, although the injected ^125^I-LECT2 might have been partially deiodinated.

### The plasma half-life of LECT2

^125^I-LECT2 was quickly eliminated from the blood within the first 10 min (Fig. 4a). The exponential curve fitting indicated that ^125^I-LECT2 had a very short plasma half-life, which was estimated to be approximately 5 min. On the other hand, ^125^I-BSA was not eliminated from the blood (Supplementary Fig. 3a) within 3 h, which is in accordance with the reported half-life of ^131^I-BSA in mice (14.5 h)^26^.

### Excretion of LECT2

The radioactivity in the kidneys reached a peak in less than 10 min after injection, followed by a rapid decrease (Fig. 4b). Conversely, the radioactivity in the urine increased 30 min after injection and remained unchanged for up to 3 h (Fig. 4c). Comparison of paper chromatography results between free ^125^I in PBS and urine sample demonstrated the presence of other than free ^125^I in the urine, although it was difficult to confirm whether the radioactivity was from the intact LECT2 protein or a decomposed peptide (Supplementary Fig. 6a). The radioactivity in the small intestinal contents also increased in a time-dependent manner (Fig. 4d). These results indicated that circulating LECT2 was excreted primarily by the kidneys into the urine and was likely excreted in bile.

To determine whether intact LECT2 passed through the kidneys to urine, urine samples from fasted C57BL/6J mice were collected for 24 h to determine LECT2 concentrations by enzyme-linked immunosorbent assay (Supplementary Fig. 6b). LECT2 was detectable in the urine samples of 24-h fasted mice but not from the ad libitum-fed mice. The urine LECT2 levels, however, were quite low (0.022 ± 0.009 ng/mL in approximately 1 mL urine) compared with the reduction in serum LECT2 to 15.2 ± 2.97 ng/mL. Furthermore, the tail vein injection of increasing non-labelled LECT2 amounts (0, 100 and 1,000 ng) led to dose-dependent increases in urinary LECT2 levels in the 24-h fasted C57BL/6J mice. These results indicated that LECT2 passed through the kidneys and excreted into the urine and that urine might contain degraded peptides of full-length LECT2 as well as the intact protein.

### Interaction of LECT2 with skeletal muscle

Our analysis identified skeletal muscle as a site that accumulated LECT2. Femoral (Fig. 5a), gastrocnemius (Fig. 5b) and extensor digitorum longus muscle (Fig. 5c), which is composed of mainly fast-twitch fibres, showed weak but significant accumulation of ^125^I-LECT2 30 min after injection (P < 0.05). Thereafter, the protein dissociated from the skeletal muscle. Conversely, soleus muscle (Fig. 5d), which is primarily composed of slow-twitch fibres, showed LECT2 accumulation in a plasma concentration-dependent manner and had the highest percentage of the injected dose per gram of tissue compared with the other skeletal muscles. On the other hand, the time-dependent tissue distribution of ^125^I-BSA in the skeletal muscles was quite different from that of ^125^I-LECT2 (Supplementary Fig. 4). These results suggest the presence of a LECT2 receptor or binding partners in all skeletal muscle types.

### Distribution of LECT2 in other tissues

A high level of radioactivity was observed in the liver tissue at 2 min after injection, which then decreased exponentially (Fig. 6a). On the other hand, a low level of radioactivity was observed at 2 min after the injection of ^125^I-BSA (Supplementary Fig. 5a). Adipose tissue is another essential insulin-targeting tissue; however, the binding of LECT2 to epididymal adipose tissue was neglectable (Fig. 6b). Similarly, no accumulation of ^125^I-LECT2 was observed in any of the tissues except the testes (Fig. 6c

## Discussion

The present study revealed that the plasma half-life of LECT2 is approximately 5 min in mice, suggesting that LECT2 does not bind to serum proteins such as albumin. Interestingly, the plasma half-life of LECT2 is very similar to that of insulin (less than 10 min)^27^. Considering the impact of obesity on many factors involved in protein kinetics, it is essential to assess parameters associated with protein kinetics in obese mice. In proteins, a short plasma half-life is usually due to a fast-renal clearance or enzymatic degradation during circulation. The rapid accumulation followed by the exponential decrease of radioactivity in the kidneys suggests that the excretion of LECT2 is mainly via the kidneys. Two factors governing protein filtration by the kidneys are the molecular size and electric charge of the protein, namely, size- and charge-selective barriers^28^. First, albumin, the most abundant plasma protein which is secreted from the liver, is approximately 65 kDa. Such large proteins are retained in the capillary lumen, reflected by the long half-life of endogenous albumin in rodents (35–39 h)^29^. Conversely, the molecular size of LECT2 is approximately 16 kDa, which can readily pass through the size-selective barrier of the glomeruli. Second, the glomerular basement membrane is a tight meshwork of negatively charged glycoproteins^30^, indicating that positively charged proteins cross the filtration barrier more readily than the negatively charged and neutral proteins. The isoelectric point values of the endogenous and recombinant murine LECT2 were 9.27 and 9.0, respectively. Furthermore, the crystal structure of human LECT2 suggests that the protein surface contains a large, positively charged region^22^. Therefore, it is reasonable to predict that LECT2 is readily excreted from the kidney glomeruli into the urine. The fast excretion through the kidneys could explain the short plasma half-life of LECT2 in the current study. Notably, the radioactivity in urine increased at 30 min after injection, which is in accordance with the LECT2 excretion mechanism proposed in the present study. It remains possible that radioactive iodide was excreted in the urine as inorganic iodide. However, paper chromatography of the urine samples showed that radiation originated from molecules other than free iodide. Thus, LECT2 protein was likely degraded, at least partially, to peptides during its excretion.

Humans have more developed cellular mechanisms for minimising protein turnover. Small proteins are readily reabsorbed from proximal tubular cells primarily by receptor‐mediated endocytosis, which aids in prolonging plasma protein half-lives in humans. Thus, the plasma half-life of albumin in humans is 20 days, which is less than 2 days in rodents^29^. Similarly, LECT2 might have a longer plasma half-life in humans compared with in mice. Further studies using human samples are necessary to determine the plasma half-life of LECT2 in humans for use as a biomarker.

The recently defined LECT2 amyloidosis has become one of the most common types of renal amyloidosis^31^. Due to its six-stranded antiparallel β-sheet structure, human LECT2 can form amyloid oligomers. Although the underlying pathogenesis remains unknown, LECT2 overexpression might be responsible for renal LECT2 amyloidosis. However, plasma LECT2 levels were within normal limits in patients with LECT2 amyloidosis^32^. The present study demonstrates that high doses of LECT2 accumulated in the kidneys, implying that increased LECT2 accumulation in the kidneys may decrease its circulating levels despite its overexpression in the liver. A single nucleotide polymorphism identified in the human *LECT2* gene leads to a codon change from ATC (A allele) to GTC (G allele) at position 172 (SNP rs31517)^33^. This codon change is accompanied with a change from isoleucine to valine at position 40 of the secreted LECT2. Because all patients with renal LECT2 amyloidosis were homozygous for the G allele, this amino acid substitution may lead to a change in its amyloidogenic conformation. Interestingly, this amino acid at position 40 is within one of the β-sheets of LECT2^22^. Our results raise the possibility that a degradation pathway might be involved in the urinary excretion of LECT2. Although the mechanism underlying LECT2 degradation remains unclear, the protein might be targeted by certain renal proteases that can recognise isoleucine but not valine at position 40. It should be noted that LECT2 itself belongs to the M23 family of metalloendopeptidases^34^, although it remains unclear whether LECT2 has a peptidase function. Therefore, the identification of the urinary peptides derived from the full-length LECT2 protein might be clinically important for the development of treatments for LECT2 amyloidosis.

Another main finding of the current study was the accumulation of LECT2 in the skeletal muscle, suggesting the presence of LECT2 receptors or binding partners in the cellular membranes of muscle tissue, specifically in fast-twitch muscles. This finding is consistent with our previous finding that LECT2 acts on the skeletal muscle to induce insulin resistance^16^. The interaction is predicted to be weak and transient. However, high levels of LECT2 in the bloodstream of obese individuals might continuously bind to the receptors, resulting in JNK phosphorylation, thereby potentially contributing to insulin resistance. LECT2 was reported to interact with three receptors, the cluster of differentiation 209 (CD209a)^12^, the receptor tyrosine kinase human c-mesenchymal- epithelial transition factor (c-Met)^35^ and the tyrosine kinase Tie1^15^. LECT2 binding to CD209a activates macrophages and is shown to have a protective effect against bacterial sepsis in mice^12^. LECT2 was also demonstrated to induce an atherosclerotic inflammatory reaction via the CD209 receptor-mediated JNK phosphorylation in human endothelial cells^36^. However, we previously reported that CD209a was not expressed in C2C12 myotubes^16^. Additionally, the Human Protein Atlas database ^37^ indicates that CD209a mRNA is not expressed in the human skeletal muscle (https://www.proteinatlas.org/ENSG00000090659-CD209/tissue). Thus, the accumulation of LECT2 in the skeletal muscle observed in the present study is unlikely due to its binding to the CD209a receptor. The binding of LECT2 to c-Met has an antagonistic effect on the Raf1/ERK signalling through protein tyrosine phosphatase 1B recruitment in hepatocellular carcinoma^35^. The HxGxD motif in human LECT2 is identified as the binding site for c-Met^35^, which is conserved in the secreted murine LECT2 protein (amino acids 35–39). The dissociation constant between human LECT2 and c-Met determined by surface plasmon resonance was 2.4 μM^22^, indicating the low affinity of LECT2 for c-Met. Assuming that radioactivity accumulation in the skeletal muscle observed in the current study was due to binding of LECT2 to c-Met, a similar accumulation should be observed in other tissues, such as the liver, because c-Met is widely expressed in a variety of tissues^38^. Similarly, LECT2 binding to Tie1 should result in accumulation in various tissues. Thus, the accumulation in the skeletal muscle observed in the current study may not be due to its binding to c-Met nor Tie1. LECT2 has an M23 metalloendopeptidase fold, and this protein family was identified to engage in protein–protein interactions in various contexts. Specifically, the crystal structure of LECT2 identified a non-conserved loop at the N-terminal region^22^, which may play an essential role in unique interactions between LECT2 and its unidentified partners. Identification of the interaction partners in the skeletal muscle is necessary to gain insights into the role of LECT2 in the development of insulin resistance. Of note, we observed increased radioactivity in the testes. Our database query in The Human Protein Atlas^37^ revealed that the most abundant expression of LECT2 protein was in the testes, whereas the *LECT2* mRNA expression was low in this tissue (https://www.proteinatlas.org/ENSG00000145826-LECT2/tissue). These results suggest the presence of an uptake receptor in the testes.

Several limitations of the present study need to be acknowledged. First, radioactivity in the thyroid was not measured; therefore, quantifying the in vivo deiodination from ^125^I-LECT2 might not have been accurate. Additionally, since ^125^I-radioactivity is detectable in any form, such as full-length LECT2, degraded peptides from ^125^I-LECT2 and free ^125^I, it is challenging to clarify the detailed mechanism underlying the urinary excretion of LECT2. A urine metabolomic approach will be useful to identify full-length LECT2 as well as specific peptides derived from its degradation. Second, the LECT2 protein was injected at a dose of 1.21 μg per mouse, which was higher than the physiological serum levels of LECT2. The dose-dependent in vivo kinetics of LECT2 remains to be elucidated. Finally, the ^125^I-labelling of LECT2 might alter its activity and/or affinity to its receptors. Future studies using other radioactive labels are needed to gain a more detailed insight into the in vivo kinetics of LECT2.

In conclusion, the present study identified that LECT2 has a very short half-life of approximately 5 min in the bloodstream in mice, indicating that LECT2 is a rapid-turnover protein with potential utility in assessing the overnutrition state. Therefore, measurement of serum LECT2 levels may be clinically useful in predicting weight cycling. In addition, there is a weak but significant interaction between LECT2 and the skeletal muscle. Identification of LECT2 receptors in the skeletal muscle will aid in advancing our understanding of obesity-associated insulin resistance.

## Methods

### Preparation of recombinant murine LECT2 and mass spectroscopy

A cDNA sequence encoding murine *Lect2* was amplified by polymerase chain reaction. The amplified fragments were cloned in-frame into the EcoRI/XhoI site of the pCMV-Myc-C vector (Clontech, Mountain View, CA, USA), in which a c-Myc tag was introduced at the C-terminus.

Murine LECT2 was expressed in Expi293 cells (Thermo Fisher Scientific, Waltham, MA, USA) in accordance with the manufacturer’s protocol. The cells were grown for 5–7 days after transfection, and the cell culture supernatant was diluted in 20 mmol/L phosphate buffer without sodium chloride and potassium chloride, pH 7.2 (buffer A) and loaded onto a HiTrap CM FF column (GE Healthcare Bioscience, Piscataway, NJ, USA) equilibrated with buffer A. After the column was washed with buffer A, the protein was eluted with a linear gradient of 0–1 mol/L sodium chloride. Next, the eluted protein was loaded onto a c-Myc-tagged protein purification cartridge (MBL International, Woburn, MA, USA) equilibrated with PBS (Wako Chemicals, Tokyo, Japan). The cartridge was washed with PBS, followed by elution with 0.5 mg/mL c-Myc tag peptide in PBS. The purified LECT2 was concentrated using VivaSpin (GE Healthcare). To determine the protein concentration, absorbance at 280 nm was measured (1 absorption unit at 280 nm = 1 mg/mL for this study). The mass spectra were measured using Ultraflex III (Bruker Daltonics, Billerica, MA, USA).

### Preparation of radiolabelled protein

The protein was labelled with ^125^I using the chloramine-T method. Briefly, ^125^I (10 MBq) and 30 μL freshly prepared 1 mg/mL chloramine-T in distilled water at room temperature were added to 100 μL LECT2 solution (approximately 5 μg) in 0.2 mol/L phosphate buffer without sodium chloride and potassium chloride (pH 7.4). Two min later, 100 μL of 1 mg/mL sodium metabisulfite was added to the solution. The radiolabelled protein was purified using a c-Myc tagged protein mild purification kit (MBL International) to remove free ^125^I, according to the manufacturer's protocol. The purity of the radiolabelled protein was determined by sodium dodecyl sulphate–polyacrylamide gel electrophoresis (CBB staining and ^125^I autoradiography). ^125^I-BSA was also prepared using the chloramine-T method. The labelled BSA was purified using the HiTrap desalting column (GE Healthcare).

### Measurement of LECT2 levels in blood and urine samples

Serum and urinary LECT2 levels were measured using the Ab-Match ASSEMBLY mouse LECT2 kit (MBL International) in accordance with the manufacturer's instructions. For measurement of urinary samples, a calibration curve was prepared ranging from 0 to 1 ng/mL of the standard LECT2 provided with the kit.

### Tissue distribution

For this study, 12–14-weak-old male ICR and C57BL/6J mice were purchased from CLEA Japan (Tokyo, Japan). After overnight fasting, the mice were injected with 0.2 mL ^125^I-LECT2 or non-labelled LECT2 solution via the tail vein and sacrificed at 2 min, 10 min, 30 min, 1 h, 2 h and 3 h by cervical dislocation. The cerebrum, cerebellum, heart, lungs, liver, gall bladder, pancreas, spleen, stomach, epididymal adipose tissue, kidney, small intestine, skeletal muscle (femoral, gastrocnemius, soleus and extensor digitorum longus muscle), femur and testes as well as samples of blood, urine and small intestinal contents were collected and weighed. The radioactivity of the samples was measured using an auto-well gamma counter (ARC-7010B, Hitachi Aloka Medical, Tokyo, Japan). The accumulation of ^125^I-LECT2 in each tissue was calculated as the percentage of the injected dose per gram of tissue. The in vivo kinetics of ^125^I-BSA was determined using the same protocol as that used for ^125^I-LECT2. ^125^I-BSA (approximately 40 kBq per mouse) was injected into overnight-fasted ICR mice. All of the mouse studies were conducted in accordance with the Guidelines on the Care and Use of Laboratory Animals issued by Kanazawa University. The experimental protocol was approved by the ethical committee of Kanazawa University (approval nos. AP-163812 and AP-194094).

### Statistical analysis

All data were analysed using the Origin 2018b software (OriginLab, Northampton, MA, USA). Numeric values were expressed as means ± standard error of the mean. Significance was compared by unpaired Student’s *t* test, and differences were considered statistically significant with P < 0.05. Curve fittings were performed using exponential or peak functions in the Origin 2018b software.

## Supplementary information

Supplementary file1.

## Data Availability

Supplementary Figure.
