# Supplementary material for: Plasma half-life and tissue distribution of leukocyte cell-derived chemotaxin 2 in mice
Source: Sci Rep. 2020 Aug 6;10:13260. doi: 10.1038/s41598-020-70192-x (PMC7411055; doi:10.1038/s41598-020-70192-x)
Supplement: Supplementary file 1 — Supplementary file1. [file 41598_2020_70192_MOESM1_ESM.pdf]

Plasma Half-Life and Tissue Distribution of Leukocyte Cell-derived Chemotaxin 2 in Mice

Akihiro Kikuchi[1, 2, *], Hiroaki Takayama[1, 3], Hirohiko Tsugane[1, 4], Kazuhiro Shiba[5], Keita Chikamoto[1, 4], Tatsuya Yamamoto[6], Seiichi Matsugo[4], Kiyo-aki Ishii[7], Hirofumi Misu[1], Toshinari Takamura[1, *]

[1]Department of Endocrinology and Metabolism, Kanazawa University Graduate School of Medical Sciences, Kanazawa, 920-8640, Japan

[2]Division of Endocrinology and Metabolism, Department of Homeostatic Regulation, National Institute for Physiological Sciences, National Institute of Natural Sciences, Okazaki, 444-8585, Japan

[3]Life Sciences Division, Engineering and Technology Department, Kanazawa University, Kanazawa, 920-8640, Japan

[4]Division of Natural System, Graduate School of Natural Science and Technology, Kanazawa University, Kanazawa, 920-1192, Japan

[5]Advanced Science Research Centre, Kanazawa University, Kanazawa, 920-8640, Japan

[6]Bioorganic Research Institute, Suntory Foundation for Life Sciences, Kyoto 619-0284, Japan

[7]Department of Integrative Medicine for Longevity, Graduate School of Medical Sciences, Kanazawa University, Kanazawa 920-8640, Japan

*Corresponding author: kikuchi@nips.ac.jp (A.K.), ttakamura@med.kanazawa-u.ac.jp (T.T.)

Supplementary Information

Supplementary Figures

Supplementary Table

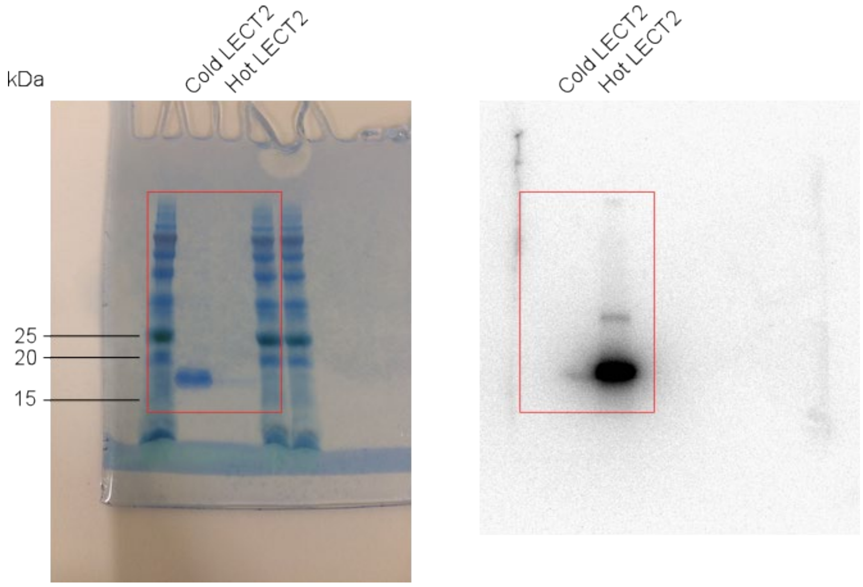

Supplementary Figure 1. Full-length gels of Figure 1 (a). Red squares represent the part of the images shown in Figure 1.

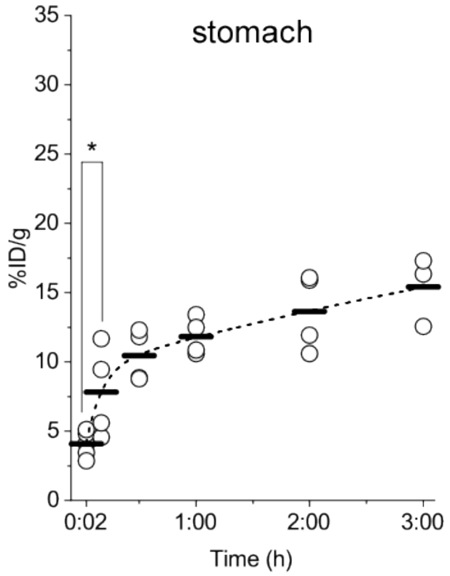

Supplementary Figure 2. Percentage of the injected dose per gram of tissue (%ID/g) in the stomach at 2 min, 10 min, 30 min, 1 h, 2 h and 3 h after ^{125}I-BSA injection. *$P < 0.05$.

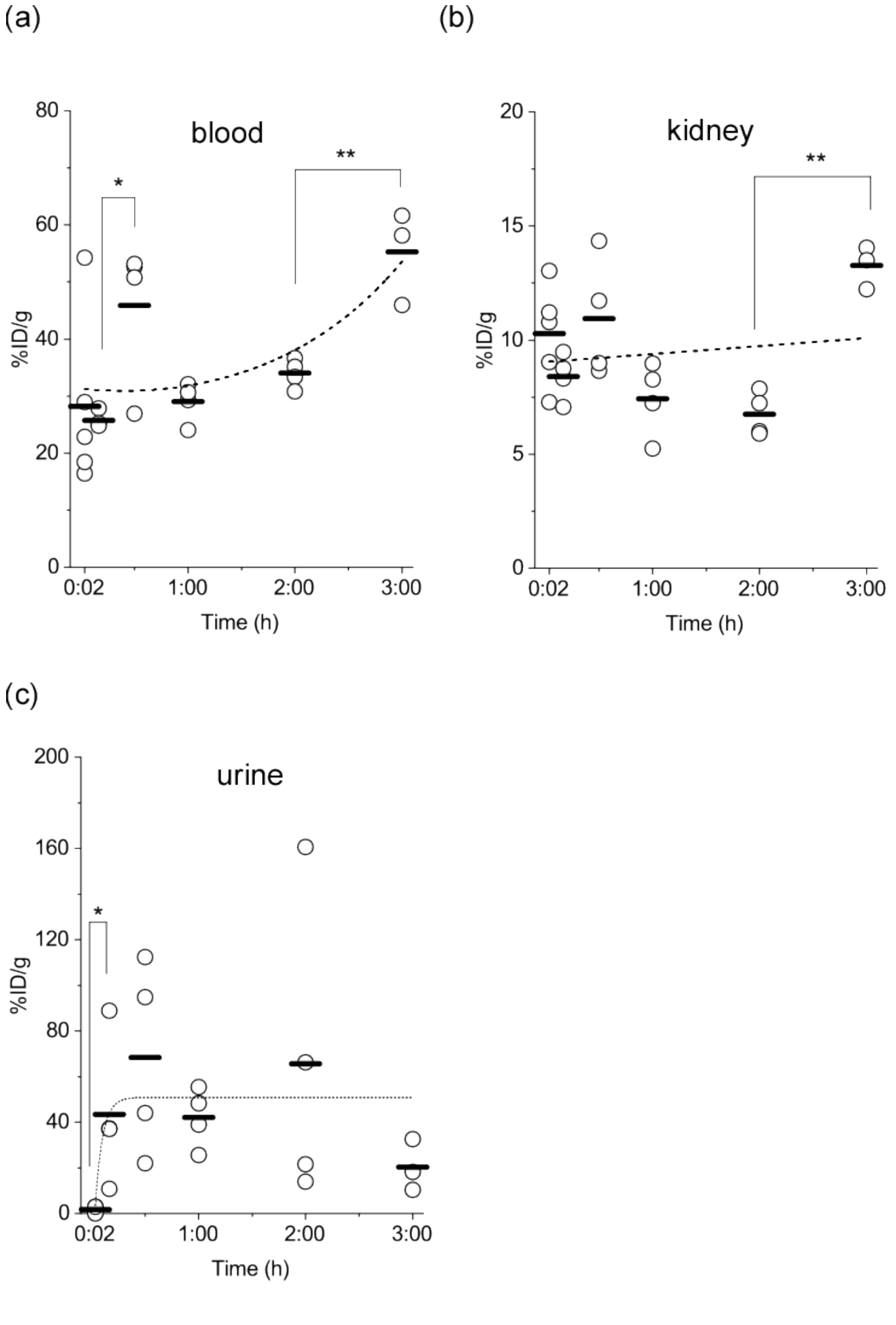

Supplementary Figure 3. Percentage of the injected dose per gram of tissue (%ID/g) in the (**a**) blood, (**b**) kidney and (**c**) urine at 2 min, 10 min, 30 min, 1 h, 2 h and 3 h after ^{125}I-BSA injection. *P < 0.05, **P < 0.01.

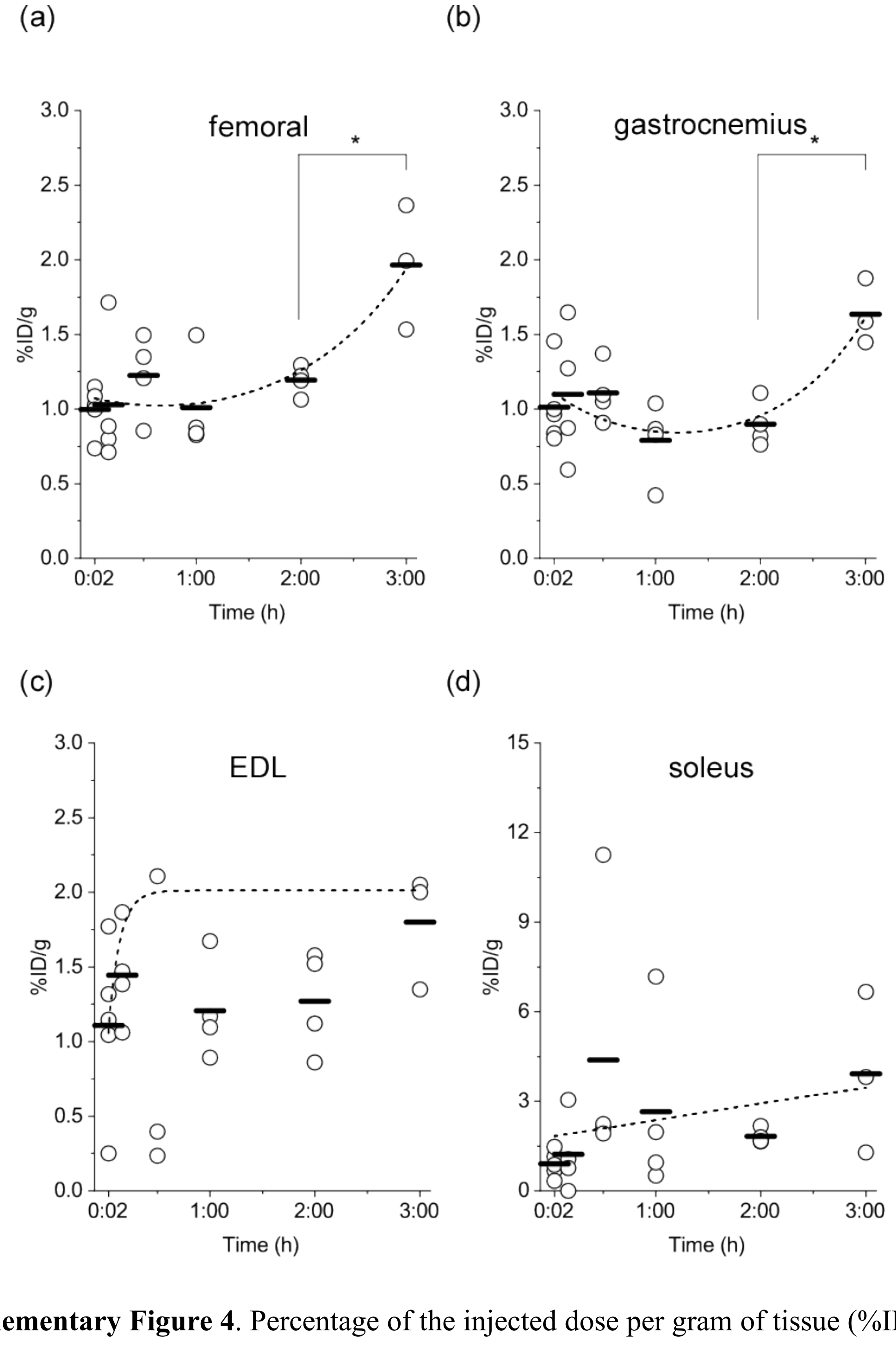

Supplementary Figure 4. Percentage of the injected dose per gram of tissue (%ID/g) in the (**a**) femoral muscle, (**b**) gastrocnemius muscle, (**c**) extensor digitorum longus muscle and (**d**) soleus muscle at 2 min, 10 min, 30 min, 1 h, 2 h and 3 h after ^{125}I-BSA injection. *$P < 0.05$.

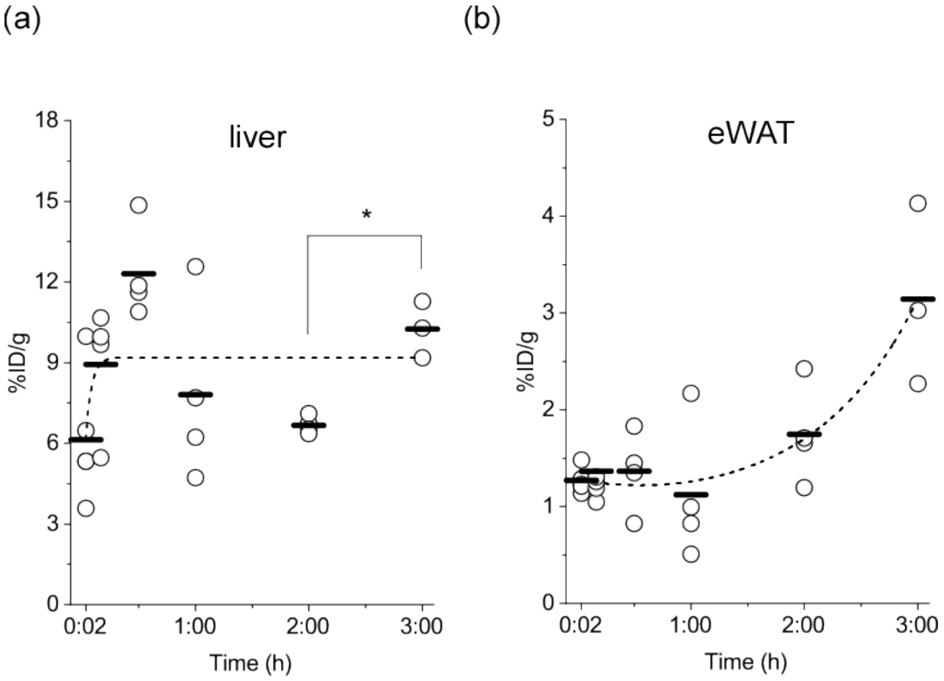

Supplementary Figure 5. Percentage of the injected dose per gram of tissue (%ID/g) in the (**a**) liver and (**b**) epididymal adipose tissue (eWAT) at 2 min, 10 min, 30 min, 1 h, 2 h and 3 h after ^{125}I-BSA injection. *P < 0.05.

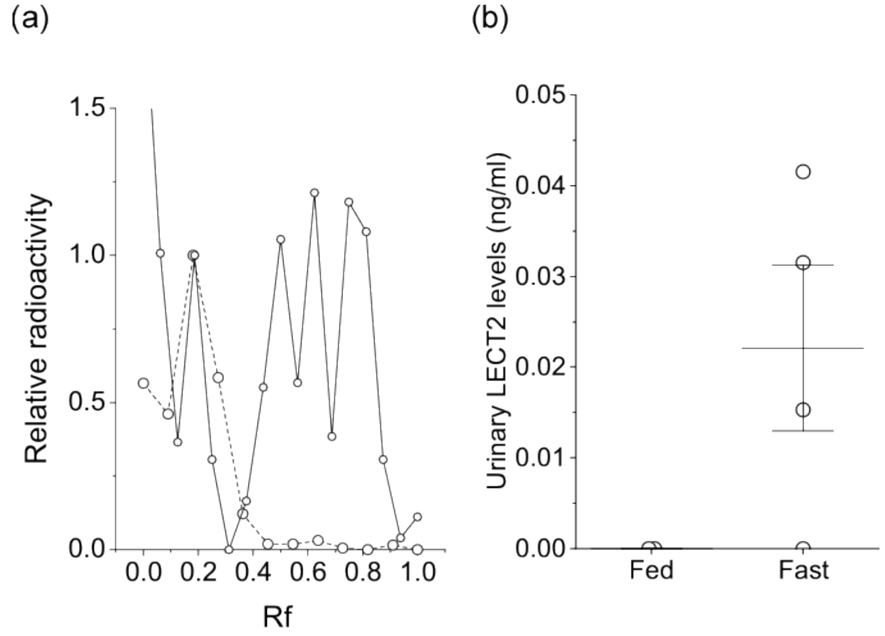

Supplementary Figure 6. (**a**) Paper chromatography analysis of radioactivity for free ^{125}I in PBS (dashed line) and urine sample collected from ICR mouse at 1 h after ^{125}I-LECT2 injection (solid line). Relative radioactivity of each Rf value is shown. PBS was used as a solvent. (**b**) Urinary LECT2 levels of *ad libitum*-fed (n = 2) and 24-h fasted (n = 4) C57BL/6J mice.

Supplementary Table 1. Percentage of the injected dose per gram of tissue in the tissues at 2 min, 10 min, 30 min, 1 h, 2 h and 3 h after ^{125}I-LECT2 or ^{125}I-BSA injection.

		**LECT2** (%ID/g)	**BSA** (%ID/g)
**Blood**	2 min	10.2	28.2
	10 min	4.8	25.8
	30 min	5.2	45.9
	1 h	3.9	29.0
	2 h	4.2	34.0
	3 h	3.5	55.3
**Kidney**	2 min	49.2	10.3
	10 min	67.7	8.4
	30 min	17.1	10.9
	1 h	6.6	7.4
	2 h	4.8	6.8
	3 h	4.0	13.3
**Urine**	2 min	0.9	1.8
	10 min	3.7	43.5
	30 min	87.5	68.3
	1 h	72.6	42.0
	2 h	113.6	65.6
	3 h	48.7	20.3
**Small intestinal contents**	2 min	0.9	-
	10 min	-	-
	30 min	3.7	-
	1 h	-	-
	2 h	6.0	-
	3 h	12.6	-
**Skeletal muscles**			-
**Femoral**	2 min	0.8	1.0
	10 min	1.1	1.0
	30 min	1.1	1.2
	1 h	0.9	1.0
	2 h	0.7	1.2
	3 h	0.5	2.0

**Gastrocnemius**	2 min	0.7	1.0
	10 min	1.1	1.1
	30 min	1.3	1.1
	1 h	1.0	0.8
	2 h	0.7	0.9
	3 h	0.5	1.6
**EDL**	2 min	0.7	1.1
	10 min	1.2	1.4
	30 min	1.4	3.9
	1 h	1.1	1.2
	2 h	0.7	1.3
	3 h	0.7	1.8
**Soleus**	2 min	6.5	0.9
	10 min	1.9	1.2
	30 min	2.0	4.4
	1 h	1.8	2.6
	2 h	1.4	1.8
	3 h	0.7	3.9
**Liver**	2 min	18.3	6.1
	10 min	12.6	8.9
	30 min	6.3	12.3
	1 h	2.3	7.8
	2 h	2.4	6.7
	3 h	1.9	10.3
**eWAT**	2 min	1.4	1.3
	10 min	0.6	1.2
	30 min	1.4	1.4
	1 h	0.6	1.1
	2 h	1.4	1.7
	3 h	1.0	3.1
**Testis**	2 min	0.7	-
	10 min	0.8	-
	30 min	1.2	-
	1 h	1.1	-
	2 h	1.6	-
	3 h	1.3	-

EDL, extensor digitorum longus muscle; eWAT, epididymal white adipose tissue
